# Eosinophile Corpuscles in Cerebrospinal Meningitis

**Published:** 1909-11-13

**Authors:** 


### SPECIAL ARTICLE.

### EOSINOPHILE CORPUSCLES IN CEREBROSPINAL MENINGITIS.

The net result of a large number of blood counts made in cases of epidemic cerebrospinal meningitis by Dr. William Dow in Glasgow* brings out two main facts about the leucocytes in this disease; first, that there is nearly always an absolute increase in the total leucocyte count, a polymorphonuclear leucocytosis, which serves to distinguish it from at least one of the other diseases—typhoid fever—for which it may be mistaken; and, secondly, that an entire absence of coarsely granular eosinophile corpuscles from the differential leucocyte count is a point of very bad prognosis.

The number of observations made was considerable. Without entering into any great detail we may give the author's own general inferences. An analysis of his observations leads him to conclude: (1) That cases of epidemic cerebrospinal meningitis are always accompanied by a leucocytosis, whether the attack is acute, abortive, mild, or chronic. (2) That the character of the leucocytosis is practically the same in all instances, both adults and children, and is the result mainly of an increase in the number of the polymorphonuclear cells. (3) That nevertheless a lymphocytosis may very occasionally be observed in infants and young children. (4) That there is a relative decrease of the large mononuclear elements alike in fatal and non-fatal cases, though less marked in the chronic type. (5) That in the first three groups there is sometimes an absolute decrease of the large mononuclear elements and occasionally total absence of these cells. In the chronic group absolute decrease, like relative decrease, is little marked. (6) That eosinophile corpuscles in acute fatal cases are always absent, although present in varying degree in all the other groups.

The highest individual count was 66,800 leucocytes per cub. mm. in an acute case. In two abortive cases the leucocytosis was observed after the condition was improving. Moreover, the number of white cells in general varies markedly from day to day and without relation to the course of the disease, nor can any clear connection be established between the leucocytosis and the character of the temperature.

It may be remarked that myelocytes were never observed in any of the films examined. Blood platelets were always present, sometimes in large numbers. No marked difference was noted in the frequency of occurrence of these elements in the fatal and non-fatal cases respectively.

As regards diagnosis, it may be again noted that the leucocytosis is only of value in excluding typhoid fever. From the point of view of prognosis, the absence of eosinophile corpuscles in the acute stage of the disease may be considered of grave significance, but it does not necessarily mean that a fatal issue will immediately ensue.

* "An investigation into the Leucocytosis of Epidemic Cerebro-spinal Meningitis." The *Lancet*, 1909, I., 829.

